# The neural correlate of mid-value offers in ultimatum game

**DOI:** 10.1371/journal.pone.0220622

**Published:** 2019-08-20

**Authors:** Xiyun Zhong, Ruojun Wang, Shiyun Huang, Jingwei Chen, Hongmin Chen, Chen Qu

**Affiliations:** 1 Center for Studies of Psychological Application, South China Normal University, Guangzhou, China; 2 College of Applied Science and Technology, Hainan University, Hainan, China; 3 Guangdong Key Laboratory of Mental Health and Cognitive Science, South China Normal University, Guangzhou, China; 4 Mental Quality Education Center, Beijing Technology and Business University, Beijing, China; Universidad Loyola Andalucia Cordoba, SPAIN

## Abstract

In the ultimatum game (UG), mid-value offers are unfair but not so unreasonable as to be rejected immediately. As a consequence, they are difficult for responders to evaluate because of the conflict that arises between two key processes, namely inequity aversion and self-interests. Since there is no clear consensus in the literature on event-related potential (ERP) as to how mid-value offers are processed, we designed an experiment to explore how the ability to reject offers influences key ERP signatures. By manipulating the right to reject offers based on game type (ultimatum game, UG or dictator game, DG), our study explored how ERPs were influenced by three types of offers available to participants (fair, unfair and mid-value). We recorded the electroencephalogram results of 28 participants while they responded to the three kinds of offers in the UG and the DG. We observed that mid-value offers in the UG elicited more negative feedback-related negativity and N400 than did the unfair offers. However, these ERP patterns were specific to the UG. Furthermore, we interpreted these results as further electrophysiological evidence of the interaction between the two processing systems during the UG.

## Introduction

The Ultimatum Game (UG) and the Dictator Game (DG) are simple bargaining games that have been studied extensively. In the UG, usually, participants are paired and must decide about how to allocate an amount of money. The first player, often called the “proposer”, decides how the money will be distributed, while the second player, the “responder”, has no choice but to reject or accept the distribution. Both know that they can allocate the money only if the responder accepts, otherwise, they receive nothing. In the DG, the responder is not given a chance to reject, the proposer’s offer while all else is the same as in the UG. Without the right to choose, the responder in the DG should have fewer opportunities to engage than in the UG. The vast majority of responders accept fair offers (5:5 or 4:6). There are two kinds of unfair offers in the UG literature: one is extremely unfair (9:1 or 8:2) and the other is more modestly unfair, and called the mid-value offers (7:3). Compared with extremely unfair offers, on which many researchers have focused, mid-value offers involve ratios that reduce norm violation and increase self-interest, which may increase the difficulty of decision-making. Previous behavioral findings show responders reject mid-value offers less often than unfair offers (9:1 or 8:2); however, participants take more time to make these decisions [1–3]. In our literature review, we found that several studies observed a trend for a larger feedback-related negativity (FRN) for a mid-value offer set to 7:3 than an unfair offer [2, 4]. These studies shared the interpretation that in the UG, mid-value offers elicit stronger cognitive conflict than do fair and unfair offers.

Out of the multiple psychological processes required, responders’ decisions in the UG comprise two separate neural systems that interact as a function of choice complexity. The dual system includes System 1 and System 2, named by Stanovich and West[5], which are responsible for fast and slow thinking, respectively. Kahneman developed the dual system theory that the operation of System 1 is fast and automatic, whereas System 2 is slower and more deliberate[6]. Moreover, System 1 is built on implicit beliefs derived from emotional experiences[7], such as inequality aversion and a preference for fairness. It is thought to make an initial evaluation of norm violation by encoding the subjective value of perceived offers [8] and signaling the appropriate negative emotional responses[9]. System 1 would theoretically “accept” a fair offer, having a 5:5 allocation scheme, because it does not violate any norms of fairness and it meets the criterion of optimal profit for the responder. Under such circumstances, the neuroimaging research has revealed activation in areas related to reward processing such as striatum and vmPFC[10–12].

Since System 1 is reflexive, intuitive, and often emotionally charged, it is also governed by habit, making it difficult to control or modify in social life, especially when conditions are computationally demanding. The responder can easily make a choice thanks to System 1 but it is hard for System 1 to deal with unfair offers. When responders receive unfair offers, their negative affective responses to unfair offers predict rejection while acceptance is also predictable because of responders’ self-interest in that any offer greater than zero is beneficial[7]. The conflict between an aversion to unfairness (norm enforcement) and self-interest (obtaining the rewards) requires a more deliberate, reflective secondary system. By integrating both social norms and economic self-interest [13–15], System 2 is thought to be capable of handling such problems flexibly via a more conscious but time-consuming route [13, 16]. Event-related potential (ERP) research of the UG has shown that unfair offers provoke more brain activity than fair offers. This pattern has been most prominently reflected by the potential named feedback-related negativity (FRN)[17, 18], which occurs over frontal brain regions approximately 240 to 320 ms after stimulus onset[19, 20]. Such a pattern has been interpreted to reflect activity related to the difficulty of decision-making in the anterior cingulate cortex(ACC ), a region considered to be part of System 2 which is thought to apply cognitive control to resolve conflicts[17]. Sanfey et al. have found greater activity in the ACC in response to unfair offers (ranging from 10% to 30% of the share) which has been interpreted as reflecting conflict detection [21–23].

As suggested by their longer latencies [1–3], mid-value offers which responders must evaluate, present a particularly good opportunity to probe the performance of both systems in decision-making. However, to the authors’ knowledge, a few studies have examined how mid-value offers influence relevant ERP and there is no clear consensus among them [2–4, 18]. A few ERP studies report that they compared mid-value offers to unfair and fair offers to investigate the additional processes required to evaluate mid-value offers occurring at different temporal stages. Polezzi et al. [2]have found a significantly large FRN for mid-value offers compared with fair offers and a larger N350 for mid-value offers than unfair offers. Meanwhile, Mussel et al[3] have reported a larger FRN in relation to mid-value offers than extreme unfair offers. Another study has observed a lower P300 for mid-value offers than for fair and unfair offers[24]. Apparent inconsistencies in ERP studies may be explained by the fact that the latter two studies introduced forms of additional experimental modulation, such as gender or expression, between the proposer and responder.

The present study used two economic games (UG and DG) with the additional manipulations of two levels of unfair offers (unfair and mid-value) alongside fair offers. In our experiment, the UG and DG were manipulated as different degrees of cognitive engagement. We expected that with more cognitive engagement, a stronger cognitive conflict would be elicited when participants played the UG rather than the DG, which would be reflected in mid-value offers. In the DG, participants would have to accept offers regardless of fairness. Thus, without having to consider the prospect of rejecting offers, participants could only perceive the fairness of the given offers. In the UG, participants would need to integrate the fairness level as well as the outcome after rejection. Each level of fairness would require different degrees of deliberation. Some researcher found that the main effect of fairness level for the P300 in the DG, with more positive responses to fair offers than unfair offers [25]. We expected the results to be more significant in N400 than N350. In our study, we asked the participants to play the UG and DG, which is more complicated, leading to a backward movement of the whole ERP waveform. Huang et al. have argued that N400 might be involved in social conflict processing. In their study, when participants’ initial ratings of the attractiveness of female faces became worse than the group ratings, the increase in conflict might have elicited more negative N400 [26]. In our study, we surmised that when participants faced offers, the stage before their offer selection would involve the FRN responsible for result evaluation. Then, during the stage of selection, participants would experience strong cognitive conflict and struggle over how to choose, which is related to N400.

We aimed to explore the different temporal processes involved in evaluation of the unfair, mid-value, and fair offers. Assuming mid-value offers would produces a stronger conflict, we expected the FRN, reflecting ACC activity, and the N400 to be larger for mid-value offers than the other ones, as in previous studies [2, 4]. When facing mid-value offers in the DG, subjects would lack cognitive engagement, and thus, the ACC might not be as activated as it would be in the UG, when participants would have decision power.

## Materials and methods

### Participants

28 students from the South China Normal University participated in the experiment (Female =15; age range = 18–26 years, M = 21 years old). They all signed informed consent, and the research was approved by the Human Research Ethics Committee of South China Normal University. All participants were right-handed, had normal or corrected-to-normal vision and had no previous experience with this kind of study. No participant reported having a history of neurological or psychiatric disorders. All were informed that they had the right to cease participation in the experiment at any point. Upon completion of the experimental procedure, participants received ¥35 (yuan; about the U.S. $5.30).

### Task

The experiment had a 2×3 within-subjects design. The first factor referred to the Type of Game (UG vs. DG), and the second factor referred to the type of offer (fair vs. mid-value vs. unfair). Consistent with the experiments by Polezzi et al., we set the fair offers as 50:50, the mid-value offers as 70:30, and the unfair offers as 90:10. Before beginning the task, participants were asked to read experimental instructions carefully to ensure a thorough understanding of the research procedure. All participants were told that two types of games should be performed; and that there would be a short break between the two games and an opportunity to familiarize themselves with the rules of the proceeding games with a practice task. Although participants were actually playing with the computer, they were informed that they would be playing with another participant, who would be in a second laboratory because of the small size of the task laboratory. Before the formal experiment began, the participants were told that they were randomly allocated to be proposers or responders to enhance credibility. In fact, they were only allocated as responders via the manufacturing of the procedure. Then we staged a mock telephone call to notify the second laboratory that it was time to start. Actually, participants were always responders. To eliminate order effects as a nuisance variable, the order of the two games was manually balanced, half of the participants played the UG first and then the DG, and the other half played in the reverse order.

**Fig 1:**
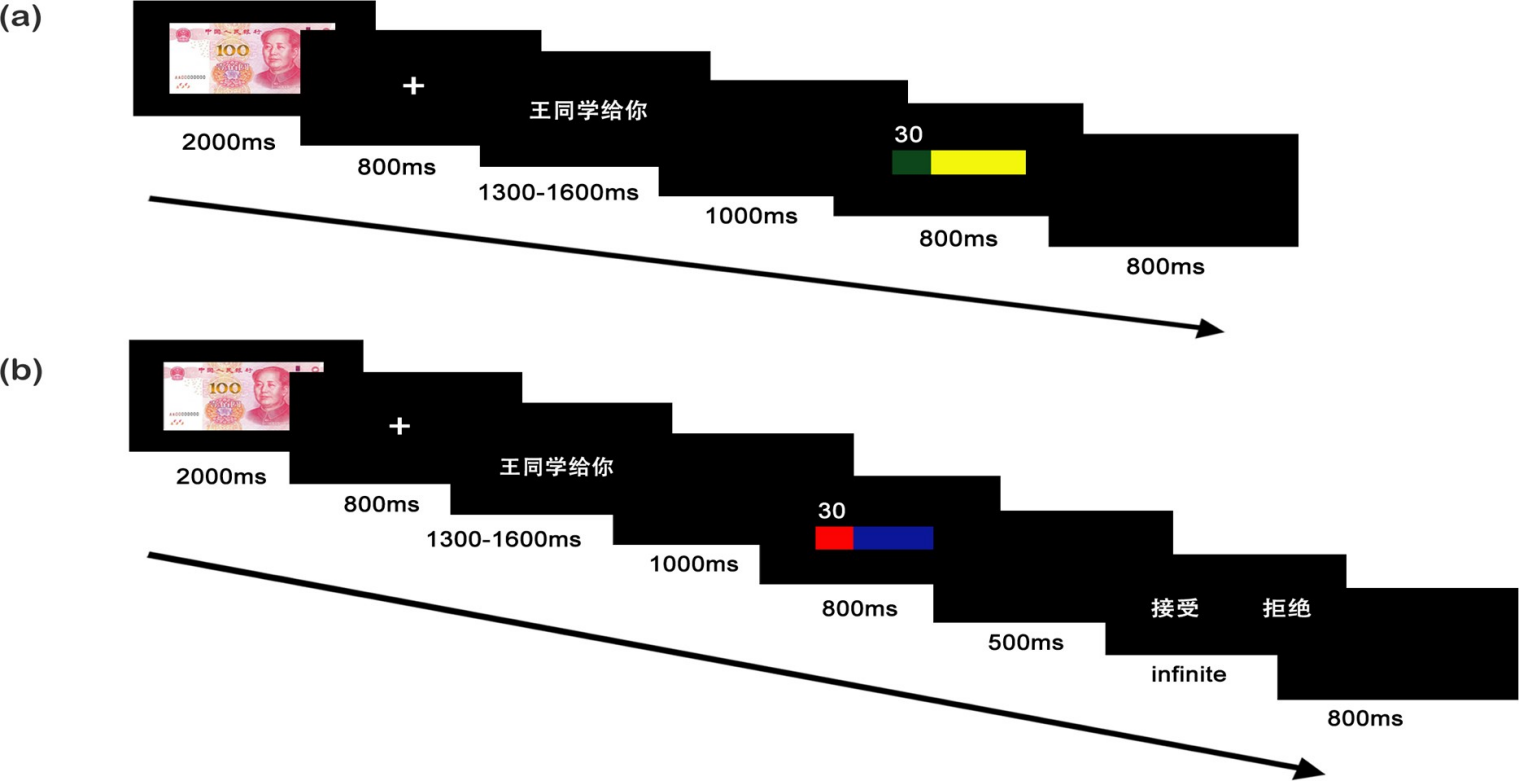
The sequence of stimuli in a single DG and UG trial (a): DG, (b): UG.

Once participants read the procedure instructions, their screens showed that they had been selected as responders. Then they were notified which game they would play first and re-presented with the rules for this game. After they finished a short practice task, the experimental task began. For example, if the UG was the first game (see **Fig 1(B)**), a single trial showed 100-yuan (about U.S. $15.10) for allocation on the screen for 2000 ms [27]. Then a white fixation lasted for 800 ms. The subsequent screen showed the phrase “Student Wang offered you” (in Chinese) for a duration ranging from 1300 ms to 1600 ms, in order to increase credibility. Between 1000 ms and the 500 ms there was a blank screen, then a horizontal bar graph appeared that showed the ratio of “offers” for 800 ms. The phrase “reject or accept” was presented to the participants, and they could press the “F” key to accept the offers or the “J” key to reject it. The response corresponding to the button was balanced among the subjects. The next trial began after a black screen that lasted 800 ms. Once the participants had completed the block, they could rest for 5s. After that, they were required to read the instructions on how to play the DG. For the DG trials, the sequence of stimuli was the same as the UG except for the “reject or accept” options because participants did not have to press a button to make a choice (see **Fig 1(A)**). To provide a more intuitive presentation, we added the figure and used various colors for the offers in the bar chart and differentiated the two games [10, 28, 29]. The participants were told that they could distinguish which game was being played (UG or DG) by the colors of the bars. In the UG, a red bar represented responders and a blue bar represented proposers; in the DG, a yellow bar meant responders and a green bar signified proposers).

The experiment consisted of 4 blocks, with each game (UG and DG) involving two blocks. A total of 300 trials were divided into 50 for each of the 6 conditions; there were three types of offers in the UG, fair 50:50, mid-value 70:30, and unfair 90:10, and there were three types of offers in the DG, fair 50:50, mid-value 70:30, and unfair 90:10. Each block consisted of 75 trials (e.g., the three types of offers in the UG were repeated 25 times in one block), which were pseudo-randomized by E-prime software. All three types of offers were randomly presented within each block. To keep the participants’ attention on the task, we added 5 trials of UG randomly occur in each block of DG, which were not included in the final statistical analysis. Once participants had completed one block, they rested for 5 s, and then they could begin another block. All four blocks were order-balanced by type of game.

To better understand how participants differentiated the pattern between the UG and the DG, we investigated predictions about offers in both games. 28 participants of the electroencephalogram (EEG) experiment were asked whether they believed they were playing with other people. Most of them reported that they answered in the affirmative and felt unfairness when faced with unfair offers. According to the Prediction Model proposed by Sanfey et al., we asked 60 participants (consisting of the 28 EEG participants and 32 additional participants, recruited in the same way, to complete a follow-up questionnaire after the experiment. The latter 32 participants answered the questionnaire but did not participate in the EEG experiment. We added them to increase the stability of the experimental result, because we wondered if the non-EEG participants’ responses might differ from those in the EEG experiment. The participants were asked to write down how many out of 100 people would allocate an offer between [0, 70] yuan (about the U.S. $0-10.6) in the UG, and then in the DG, which were also the references for the experiments in Sanfey et al.[30].

### EEG recording

EEG was recorded from 64 scalp sites at a rate of 500 Hz. All electrodes were embedded in an elastic cap. EEG signals were amplified with a bandpass of 0.01-100 Hz by the online filtering of Brain Amps (Brain Products, Munich, Germany). The online impedance between electrodes was kept below 5 KΩ. The vertical electrooculogram (VEOG) was recorded from the supra-orbital nerve of the right eye. The horizontal EOG (HEOG) was recorded from electrodes placed at the outer canthus of the left eye. We use Brain Vision Analyzer (analysis software), through an independent component analysis for continuous data, to exclude the eye-movement signal offline. After that, the EEG recordings were segmented from 200 ms before to 800 ms after stimulus onset. The Bandpass Filter was set as 0.1–30 Hz with 24db/Oct slope and at 50 Hz. We used the 200 ms pre-stimulus interval for baseline correction. EEG epochs with voltages exceeding ±100 μV were rejected. Electroencephalography (EEG) signals were acquired from the following five electrode positions: FZ, CPZ, CZ, OZ, and PZ (in relation to A1-A2 of both mastoids), which were positioned according to the international 10-20 system [31].

The analysis of ERP components included the FRN (mean amplitudes in the time window of 280-320 ms and 280–360 ms), P300 (mean amplitudes in the time window of 330-380 ms), and N400 (mean amplitudes in the time window of 360-450 ms). We conducted repeated measures analyses of variance (ANOVA) for three within-participant factors: 2 (type of game: UG, DG) * 3 (type of offer: fair, mid-value, unfair) * 5 (anterior-frontal electrodes, FZ, CPZ, CZ, OZ, and PZ). All significant effects and p-values were corrected for non-sphericity by using Greenhouse–Geisser corrections. Planned post-hoc tests were conducted for multiple comparisons when significant interactions were met.

## Results

### Behavioral results

**Fig 2:**
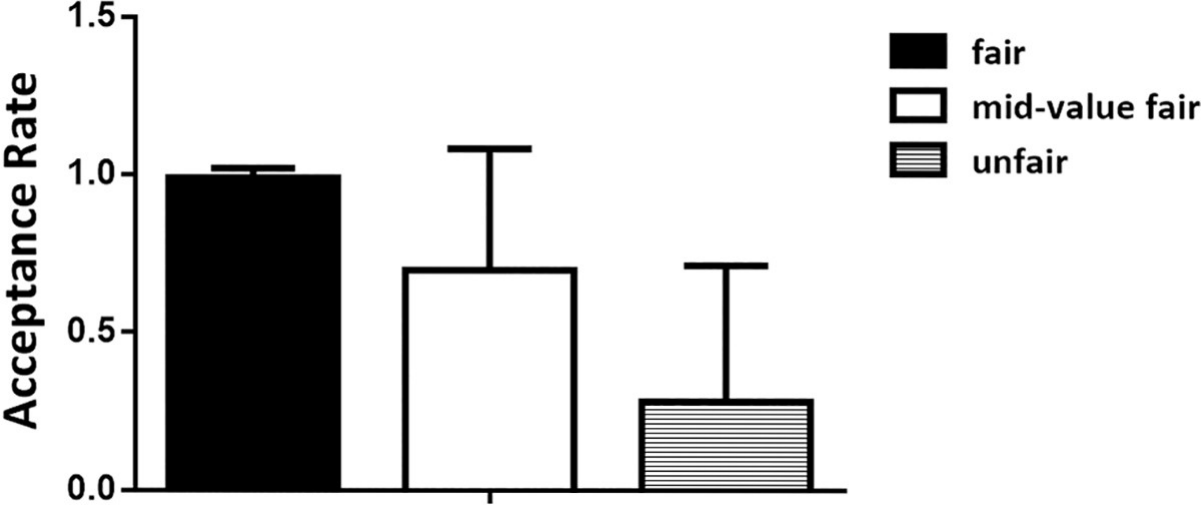
Acceptance rate for different types of offers. Error bars represent standard deviation.

Consistent with the method employed by Polezzi et al., we analyzed acceptance rates and reaction times of the UG. Our results revealed that, in the UG, the type of offer significantly influenced acceptance rates (F (2, 54) =38.732, p<0.001, η*p*2=0.589) (**Fig 2**). By using paired comparisons, as performed in previous studies [2, 4], we found higher acceptance rates for the fair offers (99.07%) than the mid-value (69.78%) and the unfair (28.18%) offers (t(54) =-8.791, p<0.001). A significant difference was found in the acceptance rates for the mid-value and fair offers (mid-value vs. fair: t(54) =-4.104, p<0.001) and in the mid-value and unfair offers (mid-value vs. unfair: t(54) =4.633, p<0.001).

After transforming the reaction time to log-value by using a logarithmic function [32], we did not find a significant difference among the types of offers (F (2, 81) = 1.670, p=0.195). Post multiple comparison test revealed that the time it took to decide about mid-value offers (M=588.08±298.25ms) was longer than it was for fair (M=466.27±172.71ms, t (54) =-1.870, p=0.067) and unfair offers (M=552.83±280.72ms, t (54) =-1.390, p=0.170). We considered the possible reasons subjects did not react immediately. Our task was set that the “offers” screen showed for 800 ms and a blank screen for 500 ms, so responders had to decide after 1300ms, which led to inaccurate reaction times.

We used the questionnaire results completed by 60 subjects to compare how predictions changed with the function of the type of game. According to the model prediction, those participants’ predictions could create a distribution of the frequency of offers and establish a linear contrast of expectations. Based on Sanfey et al[33], we calculated the weighted mean of this distribution to represent each participant’s initial expectation. For instance, if a subject reported that he or she would expect 20 offers of ¥50 (about U.S. $7.55), 20 offers of ¥40 (about U.S. $6.04), 40 offers of ¥30 (about U.S. $4.53), and 10 offers of ¥20 (about U.S. $3.02), then their calculated expectation amount per offer would be ¥ 32 (about U.S. $4.83); that is, (20 × ¥50) + (20 × ¥40) + (40 × ¥30) + (10 × ¥20) /100. Thirty-two yuan would be regarded as the mid-value offers. The statistical results revealed that participants thought 66.67% of 100 participants would allocate a fair offer in the UG. In addition, they believed 15% of 100 people would allocate the fair offers in the DG. They felt that, in the DG, more participants would choose mid-value offers (58.33%) than fair and unfair offers (26.67%).

### EEG data

#### FRN

**Fig 3:**
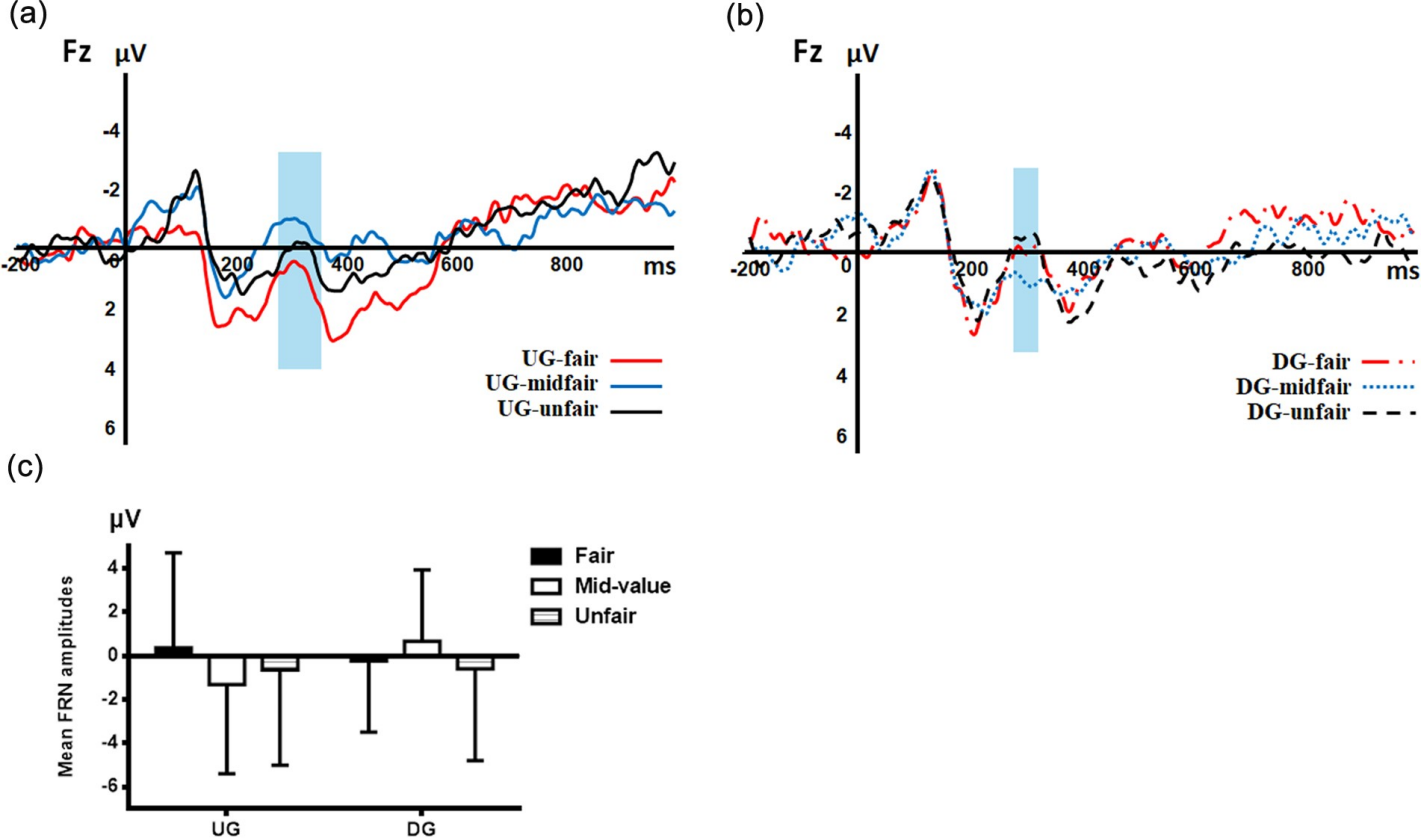
Grand average event-related potentials at FZ. (a) Grand average event-related potentials at FZ for UG in the 280-360 ms time window; (b) Grand average event-related potentials at FZ for DG in the 280-320 ms time window; (c) Bar chart of FRN mean amplitudes for six types of experimental conditions at FZ. Error bars represent standard deviation.

According to the whole waveform, the FRN in our study was analyzed in the 280-360 ms temporal window for the UG and 280–320 ms temporal window for the DG (see **Fig 3 (A)** and **Fig 3 (B)**), because the UG and DG were different games, and the ERP component might occur at different times under different conditions [34] . An ANOVA, consisting of type of offer (fair, mid-value, unfair), type of game (UG, DG), and electrodes position (CPZ, CZ, FZ, OZ, PZ), revealed a significant three-way interaction (F (8,216) =4.808, p=0.003, η*p*2=0.151) with a significant main effect of electrodes position (F (4,108) =14.992, p<0.001, η*p*2=0.357). Post-hoc analyses showed a more negative FRN at FZ electrodes (-0.287±3.495μV) than the others (CPZ: 4.458±3.64μV, p<0.001; CZ: 2.806±3.645μV; p<0.001; OZ: 2.884±4.565μV, p<0.001; PZ: 5.395±3.801μV, p=0.021) [17, 28, 35]. However, neither the main effect of type of offer (F (2, 54) =0.435, p=0.650, η*p*2=0.016) nor type of game (F (1, 27) =0.104, p=0.749, η*p*2=0.004) was significant. There was no statistical significance for the interaction effects of electrode position and type of game (F (4, 108) =1.997, p=0.151, η*p*2=0.069), electrode position and type of offer (F (8, 216) =2.165, p=0.095, η*p*2=0.074), as well as type of game and type of offer (F (2, 54) =2.040, p=0.140, η*p*2=0.070) (See **Fig 3(C)**).

To analyze the simple effect of the three-way interaction, we conducted a two-way ANOVA at FZ. The interaction of type of offer and type of game was significant (F (2, 54) =8.486, p=0.001, η*p*2 =0.239), suggesting the FRN patterns were different between the DG and the UG. Specifically, pairwise comparisons showed a more negative FRN for mid-value offers than fair (p=0.002) and unfair (p=0.016) offers in the UG. We also found a larger FRN for unfair offers than fair offers (p=0.039) in the UG. When participants played the DG, the FRN in the 280-320 ms temporal window was larger for unfair offers than mid-value offers (p=0.034). However, we did not find a significant difference for fair offers compared with mid-value (p=0.153) or unfair offers (p=0.530). For the mid-value offer condition, an enhanced FRN was elicited during the UG, in comparison with the DG (p=0.002). We did not observe such a difference between the UG and DG performances for either unfair (p=0.281) or fair offers (p=0.915). But the main effects of type of game (F (1, 27) =1.584, p=0.219, η*p*2 =0.055) and type of offer were not significant (F (2, 54) =1.565, p=0.218, η*p*2 =0.055).

#### P300

**Fig 4:**
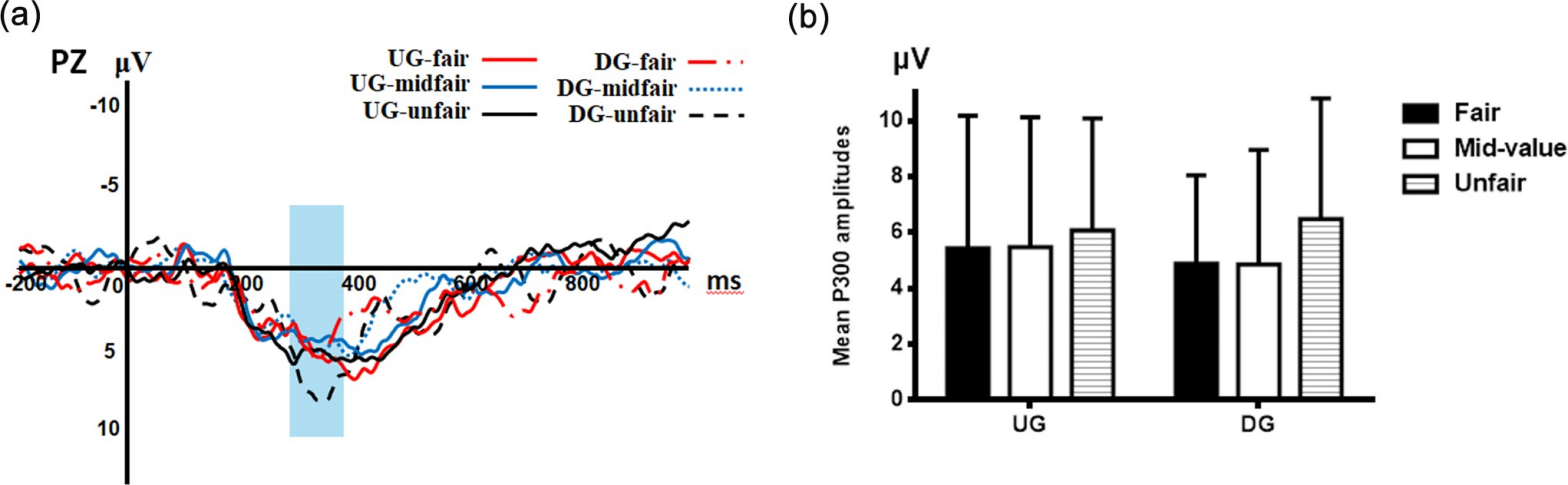
(a) Grand average event-related potentials at PZ; (b) Bar chart of P300 mean amplitudes for six kinds of conditions at PZ. Error bars represent standard deviation.

A 3×2×5 ANOVA explored the mean amplitudes in the 280-380 ms time window (see **Fig 4(A)**), and revealed a significant three-way interaction (F (8,216) =3.608, p=0.011, η_*p*_^2^=0.118) and a significant main effect of electrode position (F (4,108) =13.783, p<0.001, η_*p*_^2^=0.338). Post-hoc analyses showed a more positive P300 at PZ electrodes (5.538±3.531μV) compared with other electrodes (CPZ:4.732±3.402μV, p=0.021; CZ:3.078±3.556μV, p<0.001; FZ: 0.013±3.674μV, p<0.001; OZ:2.885±4.598μV, p=0.001)[27]. However, neither of the main effects of type of offer (F (2, 54) =1.281, p=0.286, η*p*2=0.045) and type of game (F (1,27) =0.001, p=0.976, η*p*2<0.001) nor the interaction effects of electrode position and type of game (F (4, 108) =2.474, p=0.096, η*p*2=0.084), electrode position and type of offer (F (8, 216) =2.569, p=0.056, η*p*2=0.087),type of game and type of offer (F (2, 54) =2.653, p=0.080, η*p*2=0.089) reached statistical significance.

To analyze the simple effect of the three-way interaction, we conducted a two-way ANOVA at PZ and found a significant main effect of type of offer (F (2, 54) =3.205, p=0.048, η*p*2=0.106). Importantly, the post-hoc pairwise comparison showed an enhanced P300 positivity during the DG for unfair offers compared with mid-value (p=0.045) and fair offers (p=0.020), while the difference between fair and mid-value offers was non-significant (p=0.957). We did not find a similar effect in the UG condition (See **Fig 4(B)**). Moreover, when we compared mid-value offers with fair (p=0.956) and unfair offers (p=0.221), the fair offers compared with unfair offers (p=0.249) did not reach statistical significance. P300 did not show a significant difference from the UG compared with the DG for fair (p=0.312), mid-value (p=0.349) or unfair offers (p=0.536). However, we found the main effect of type of game (F (1, 27) =0.270, p=0.608, η*p*2=0.010) and a significant interaction effect (F (2, 54) =1.631, p=0.205, η*p*2=0.057) without significance.

#### N400

**Fig 5:**
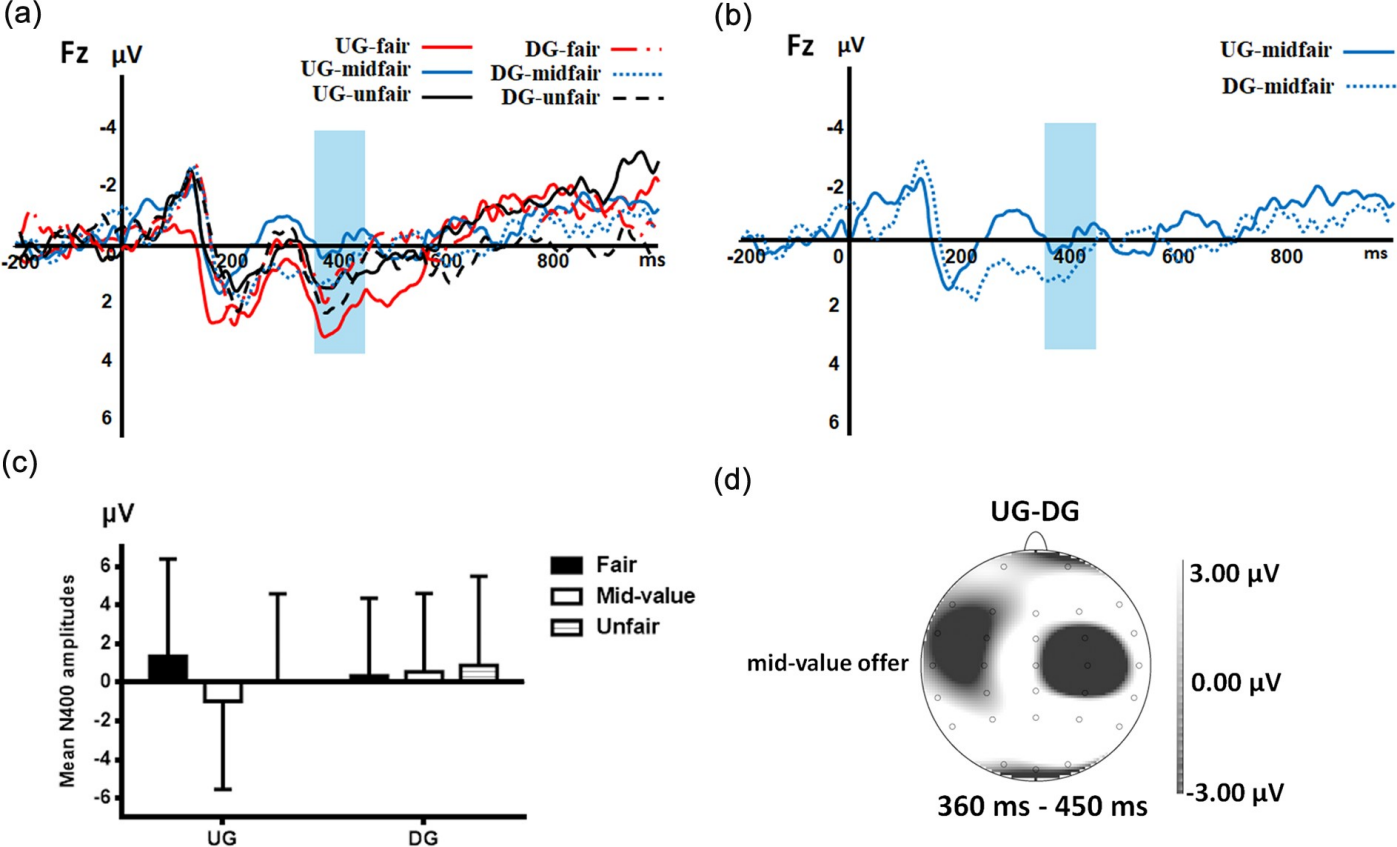
(a) Grand average event-related potentials for six types of conditions at FZ; (b) Grand average N400 event-related potentials for the mid-value offers at FZ; (c) Bar chart of mean amplitude for six types of conditions for N400 at FZ; Error bars represent standard deviation. (d) Topographic map of N400 in the 360–450 ms time window for the mid-value offers.

A 3×2×5 repeated measures ANOVA exploring mean amplitudes in the 360-450 ms time window (See **Fig 5(A)**), revealed a significant three-way interaction (F (8,216)=2.948, p=0.024 η_*p*_^2^=0.098), with the significant main effect of electrode position (F (4,108)=9.647, p=0.002, η_*p*_^2^=0.263). Post-hoc analyses showed a larger N400 at FZ electrodes (0.362±3.988μV) compared with other positions (CPZ: 4.686±3.198μV, p<0.001; CZ: 3.212±3.620μV, p<0.001; OZ: 2.746±4.895μV, p<0.001; PZ: 5.104±3.423μV, p=0.111). We also observed the significant main effect of type of game (F (1, 27) =5.005, p=0.034, η_*p*_^2^=0.156), while type of offer did not reach significance (F (2, 54) =1.370, p=0.263, η_*p*_^2^=0.048). The interaction effects of electrode position and type of game (F (4, 108) =6.083, p=0.008, η_*p*_^2^=0.184), electrode position and type of offer (F (8, 216) =2.688, p=0.045, η_*p*_^2^=0.091), type of game and type of offer (F (2, 54) =5.961, p=0.005, η_*p*_^2^=0.181) were significant.

We also conducted a two-way ANOVA at FZ to analyze the simple effect of the three-way interaction. The interaction effect of type of offer and type of game at FZ electrodes was significant (F (2, 54) =9.122, p<0.001, η*p*2 =0.253). Post-hoc pairwise comparisons revealed that, in the UG, the N400 at FZ electrodes showed greater negativity for mid-value offers than fair (p<0.001) and unfair (p=0.026) offers. We also noticed that the N400 was greater for unfair offers than fair offers (p=0.020). We did not observe any significant differences during the different offers when participants played the DG (See **Fig 5(B))**. The mid-value offers compared with fair (p=0.648) and unfair offers (p=0.573), as well as fair offers compared with unfair offers (p=0.402) did not reach statistical significance. On the other hand, for mid-value offers, a larger N400 was elicited from the UG compared with the DG (p=0.045). However, these effects were not found for fair (p=0.074) and unfair offers (p=0.102). (See **Fig 5(C)** and **Fig 5 (D)**). We did not find statistical significance for the main effects of type of game (F (1, 27) =0.892, p=0.353, η*p*2 =0.032) and type of offer (F (2, 54) =3.064, p=0.055, η*p*2 =0.102).

## Discussion

Consistent with basic predictions, we found that when responders played the UG, they tended to accept fair offers and reject unfair offers. Also, as predicted, the acceptance rates for mid-value offers were lower than those for fair offers but higher than those for unfair offers. At the ERP level, we observed a pattern in which there was a greater FRN for mid-value offers than for fair and unfair offers in the UG. However, the greater FRN effect for mid-value offers disappeared in the DG. Nonetheless, the FRN evoked by unfair offers were larger than those evoked by mid-value offers in the DG. In addition, the P300 showed greater positivity for unfair offers than for the other types of offers when participants played the DG, which was not found in the UG. We observed that the N400 pattern when participants played the UG, more negative N400 was elicited by mid-value offers than the two other types of offers, which we failed to find in the DG.

Our behavioral data revealed that, in the UG, the acceptance rates of the three offers were distinct, which meant that the offers differed in the participants’ perceptions. The acceptance rates of mid-value offers were significantly lower than those of the fair offers, and higher than those of the unfair offers, which was consistent with the findings of previous studies [3, 18]. We argued that the acceptance rates of the mid-value offers would be situated in the middle between fair and unfair offers, as a kind of reconciliation product between the processes induced by those extreme offers. We expected the responders to experience internal conflict between choosing to punish the proposer by rejecting their offers and following self-interest by taking the money.

We found that unfair offers evoked more negative FRN than fair offers, which slightly different from the results of Polezzi et al.[2], that is, we found that unfair offers evoked more negative FRNs than fair offers. We observed greater FRN for mid-value offers than the other two offers when the participants played the UG, which was in line with previous studies [28, 35]. Meanwhile, in the DG, the FRN evoked by mid-value offers appeared to be more positive than that of the fair and unfair offers. Different outcomes for both games meant different patterns for them. We speculated that the reason for the difference between the two patterns might be the different expectations of participants about the two games. Therefore, we administered a follow-up questionnaire to 60 participants. In this post-hoc test, participants predicted that among 100 people, when they played the UG, about 66.67% of proposers would make a fair offer, whereas 85% of proposers would make a less fair offer when playing the DG. They expected more participants to propose unsatisfactory offers in the UG than the DG, because responders had no choice in the DG. That was why responders in the UG showed more negative FRN in mid-value and unfair offers than the fair offers; they thought they deserved a fairer offer in the UG[36]. According to questionnaire results, the pattern of the DG was that the responders, who had lower expectations, were better able to accept mid-value offers, which differed from the UG pattern.

Patterns of FRN responsibility had been interpreted in several ways. It had been argued that FRN might represent a reward prediction error [37, 38]. However, several studies suggested that FRN was associated with unfavorable outcomes, such as incorrect responses and monetary losses [39, 40]. This association reflected a more general interpretation that involved a rapid evaluation of events along with an abstract good-bad dimension in which a negative outcome would elicit a larger FRN [37]. In addition, some researcher suggested that the FRN magnitude was a deviation from expected value rather than a representative of the absolute magnitude of reward[41] and that it reflected the detection of conflict between expectancy and actual outcomes [39]. Thus, unexpected offers, rather than simply unfair offers, elicited a large FRN. Consistent with expectation theory, we found that in the UG, when participants expected fair offers, the unfair and mid-value offers induced larger FRN. In the DG, the prediction of participants was close to the value of mid-value offers, which led to a smaller FRN than that evoked by the unfair offers. And the fair offers might be influenced by expectancy and passive emotion, which seem to elicit a larger FRN than mid-value offers but did not reach statistical significance. These results were caused by the difference in participants’ predictions of offers between the UG and the DG.

We found a greater FRN produced by a stronger cognitive conflict when the responders were allocated the mid-value offers in the UG. However, thus far, no conclusion has been made yet on mid-value offers in the UG [2–4, 18, 42]. A similar experiment, which was designed by two sets of researchers, had different results. One researcher used the six types of offers(distribution ratio: 11:1, 10:2, 9:3, 8:4, 7:5, 6:6) in the UG and found a linear trend with an increasingly negative FRN with an increasing level of unfairness. However, the unfair offers (10:2) were accepted by responders in about 50% of trials, which was the highest variance in the decision process among all the offers. In terms of in P300, a minimum of the mid-value offers (9:3) was found [18]. Another study applied eight kinds of offers(8 levels, 11:1 to 4:8) and found that FRN amplitude was more negative for the mid-value offers (9:3) than the fair (6:6) and unfair (11:1) offers[3]. Nonetheless, other studies have observed a trend that unfair offers elicited a larger FRN than the mid-value offers set to 7:3 without insignificance[42]. These studies all shared the interpretation that the mid-value offers elicited stronger cognitive conflict than the other two offers in the UG. In our opinion, the influence factor for the different experimental results might be the lack of a clear definition between the mid-value offers and the other offers. Because the mid-value offers were context-dependent, the feeling toward the mid-value offers might change as the extreme offers of the UG change. For example, in the two experiments above, compared with the extremely fair offers of 6:6, the responders might confuse which one (9:3 or 8:4) was a mid-value offers; not to mention that the different research might set the extremely unfair offers as 10:2. That might lead to a cognitive conflict elicited by mid-value offers that were not strong enough [43, 44]. Furthermore, most studies did not specifically explore the mid-value offers, which might be another reason for these instabilities in the ERP results. In our experiment, we designed three types of offers to specialize in mid-value offers, which was obviously different from the fair and unfair offers. By this mean, we found that mid-value offers in the UG indeed evoked a greater FRN than the fair and unfair offers as well as the mid-value offers in the DG. Findings were consistent with the model prediction of Sanfey et al., showing that the responders would be more likely to decline the mid-value offers when they had higher instinct prediction. Otherwise, the participants with higher expectations were associated with increased activity in the ACC when receiving the mid-value offers [30]. Therefore, we argued that the mid-value offers in the UG evoked more cognitive conflict because more negative FRN was induced.

Responders regarded mid-value offers differently in the UG and DG. With the right to choose in the UG, they had high expectations and more complex decision-making processes. Unlike the UG, they thought less about how to make decisions and focused on self-interest and the feeling of inequity without option. Different patterns of FRN were found between the UG and DG, especially for the mid-value offers. We considered the mid-value offers was a good example for exploring the interaction between the System 1 and System 2. We assumed that when a participant was receiving a fair offer in the UG, which would be the optimal profit, System 1 would dominate making decisions[8, 45], which might be reflected by positive FRN in our experiment. The dorsal anterior cingulate cortex (dACC), as a key region of System 2 plays an important role in conflict monitoring[46, 47]. Feng et al pointed out that receiving unsatisfactory offers evoked stronger cognitive conflict by the dACC than fair offers in the UG [16]. Moreover, some researcher illustrated that the intuitive response conflict with self-interest caused a conflict signal encoded by the dACC, which might activate cognitive control from the reflective, deliberate System 2 to resolve the conflict instead of suppressing the intuitive responses of System 1[7, 16, 48]. We assumed that the mid-value offers, which would elicit a larger conflict, would require longer reaction time and more involvement from System 2. In keeping with our assumption, we found that the mid-value offers were associated with a larger FRN than fair and unfair offers in the UG. In contrast, the mid-value offers in the DG, without decision-making and cognitive conflict, evoked a smaller FRN than in the UG. We suspected that mid-value and unfair offers might evoke different degrees of conflict, which might cause different degrees of involvement from System 2 in the decision-making process. With cognitive engagement, System 2 might play a part in the decision-making process but not function as a dominant system in unfair offers. When faced with mid-value offers, participants showed stronger cognitive conflicts, which were accompanied with more negative FRN and more activation of ACC; thus, the complex decision-making required a more conscious system, the deliberate System 2, rather than the intuitive System 1. At this point, System 2 might be the dominant in the decision-making process when participants were receiving mid-value offers in the UG. We believed that from simple to complex decision-making, such as from fair to mid-value offers in the UG, there might be a process of dominant system shifting from System 1 to System 2. Those findings provided further electrophysiological evidence for this argument.

We also examined the N400, during the window of 360-450 ms. Our findings were similar to those for the FRN; a greater negative amplitude was found for mid-value offers in the UG than that elicited by them in the DG. The pattern of N400 could be generated by non-semantic conflict [49], such as cognitive conflict or emotional conflict. Chen et al.[50] argued that the N400 was related to conflict, which meant a larger conflict would evoke more negative N400 amplitude. According to these results, since mid-value offers in the UG induced stronger cognitive conflict, they would also elicit a more negative N400 than fair and unfair offers. However, when participants played the DG, they did not experience cognitive conflict because of a lack of cognitive engagement, which led to smaller N400 amplitude than that in the UG. These N400 findings, from another point of view, also provided electrophysiological evidence of a mid-value offer’s inducing stronger cognitive conflict in the UG. Consequently, we found that the mid-value offer’s FRN and N400 were more negative than those of fair and unfair offers, which might prove to be an advantageous transformation of the decision-making systems in the face of the three types of offers in the UG. Combined with the experimental results, we thought the evaluations of the mid-value offers led to a stronger FRN of the mid-value offers than that of the fair and unfair offers in the UG. Since FRN reflects the subjective value of outcomes, it evaluates outcomes with multiple dimensions[51]. For example, in the UG, accepting a mid-value offer means more money with negative feelings caused by unfair aversion, while rejecting it means less money with better feeling due to revenge. Double conflicts in decision-making make mid-value offers more troublesome than other offers. In particular, when faced with a mid-value offer in the UG, participants experienced more complex considerations and greater cognitive conflict, which might be why mid-value offers induced larger FRN than unfair offers. In the DG, without cognitive engagement, the mid-value offers elicited different degrees of N400. Certainly, further research is needed for more in-depth discussion.

When participants played the DG, we found more positive P300 amplitude for unfair offers than for fair offers. We thought these results relate to valence, as defined in terms of high-level effective evaluations, such as disgust or disappointment, rather than straightforward reward values[52]. The results were also consistent with seminal work on decision-making which showed that losses loom larger than gains [53] and indicated that the arousal elicited by a loss of a certain magnitude, was greater than the arousal elicited by a gain of the same magnitude. In our study, for the participants, unfair offers meant a loss due to the less benefit, and fair offers might have stood for gain.

There were several limitations to our study. First, we acquired an inaccurate reaction time because the subjects could not directly react when they saw the horizontal bar graph showing the ratio of “offers”. They had to wait through the 800 ms of the “offers” presentation and 500 ms of the blank screen. In other words, within 1300 ms of seeing the “offers”, the subjects could start thinking about what to choose, but they could not press the button to respond. This setting was to prevent the subjects’ keystroke selection from the extraction of EEG data, but it resulted in unsatisfactory behavioral results. After collecting and analyzing the statistics, our data showed that there were many short reaction times (about 100-200 ms) in the selections, indicating that the participants made their decisions before pressing the button, and the data of the reaction time was not of much reference value. Second, in this experiment, we did not evaluate participants’ personality traits or psychological states, which may have influenced on the results of the experiment, especially those with extremely positive or negative valence. In addition, some researches has found that responders who are more prone to self-interest experience larger conflict and stronger responses in the dACC[54, 55]. Depression, social anxiety, and empathy have been shown to modulate ERPs related to emotional processing. Therefore, the subjects’ attitudes toward money and emotional appraisal [13, 56] might be explored further in future studies.

## Conclusion

This study explored the different temporal processes involved in evaluating unfair, mid-value, and fair offers and investigated whether cognitive engagement in decision-making influenced the temporal process of evaluating different offers. Our study is the first to focus on how decision-making systems evaluate mid-value offers in the UG and DG. Because of this manipulation, we observed distinct different ERP patterns. We found that when participants played the UG, the mid-value offers, which were associated with increasing cognitive conflict and engagement, evoked greater negativity in FRN and N400 than did the unfair and fair offers in the UG and the mid-value offers in the DG. We argued that these ERP findings illustrated increasing cognitive conflict and subsequent growth in the involvement of System 2 in decision-making.

## Supporting information

S1 Fig Supplemental Data of UG and DG (Doi: 10.6084/m9.figshare.9037808). (XLSX)
